# Productivity analysis of regional-level hospital care in the Czech republic and Slovak Republic

**DOI:** 10.1186/s12913-022-07471-y

**Published:** 2022-02-11

**Authors:** Ivana Vaňková, Iveta Vrabková

**Affiliations:** Department of Public Economics, Faculty of Economics, VSB – Technical University of Ostrava, Sokolská třída 33, 702 00 Ostrava 1, Czech Republic

**Keywords:** Data envelopment analysis, Hospitals, Malmquist index, Regional disparities, Window analysis

## Abstract

**Background:**

Providing hospital care is an essential objective of national health policies. The countries that share common history, when they emerged from the same health system and similar conditions in the early 1990s, after the division of Czechoslovakia, became the objects of evaluation of the development of technical efficiency of hospital care. The subsequent development of their health care system also was very similar, but no longer entirely identical. The article aims to identify the trends and disparities in the productivity of the capacities of hospital care on the regional level (NUTS III.) in the Czech Republic and the Slovak Republic in 2009–2018 before the COVID-19 pandemic using the multi-criteria decision methods.

**Methods:**

The window analysis as a dynamic DEA method based on moving averages and also the Malmquist Index, that allows the evaluation of changes in relative efficiency and of changes in the production possibilities frontier have become the key methods for evaluating the over time efficiency evolution. To model technical efficiency, an output-oriented method assuming constant returns to scale was chosen. Aggregated input and output parameters for each region were the object of study.

**Results:**

The results showed that differences in the efficiency trends in terms of the examined parameters among the individual regions are slightly greater in the Czech Republic than in the Slovak Republic. The least efficient regions are those where capital cities are located. Furthermore, the analysis showed that in 2018 all of the Slovak Republic regions improved its productivity compared to 2009 and that technological conditions had a significant impact on this improvement. The results of the Czech Republic regions show productivity improvement in 57% of the regions that, on the contrary, was due to changes in technical efficiency.

**Conclusions:**

It should be recommended to the state- and regional-level governments to refrain from unilaterally preferring the orientation of public policies on the efficiency of the provision of hospital care, and rather focus on increasing the quality and availability of hospital care, especially in smaller, rural, and border regions, in the interest of population safety during pandemics and other emergencies.

**Supplementary Information:**

The online version contains supplementary material available at 10.1186/s12913-022-07471-y.

## Background

Forming and implementation of healthcare policies is a dynamic process aimed at delivering corresponding solutions to medical problems within the society. The common interest is always the health of the population, which may, at a certain point, depend on the quality of healthcare. Němec et al. [1] evaluated the healthcare policies in the CR in the period from 1990 to 2009, outlining seven partial stages in the transformation of the Czech healthcare system. The Czech example confirms the limited potential of economic competition within the Czech healthcare system, as well as the fact that the efforts for liberalisation have been linked, for a larger part, to the early stages of the healthcare system, a fact that is also true of other EU countries. In contrast, the issue of efficiency and efficacy of the healthcare system has remained a topical problem and the aim of the healthcare policies, one that has to react to quickly changing conditions.

The current healthcare policy in the Czech Republic within the “Health 2030” strategic framework pursues, among other things, personal and technological stabilisation of healthcare. White areas in the capacities of healthcare services, mainly the insufficient number of skilled professionals, are perceived prominently and differently with respect to medical professions and regional conditions, [2]. In the conditions of the Slovak Republic, by contrast, healthcare policies put increased emphasis on the efficiency of provision of healthcare and implementation of the Value for Money approach, making it their priority and programme, [3].

The World Health Organisation’s strategy (WHO, [4]) points out that the essence of management at all levels of the system is healthcare. In terms of the macro-level, this involves application of measures aimed at setting the system of regulations, especially the monitoring of the volume of services. Essential objectives include ensuring the availability, efficiency, and quality of the healthcare provided. At the micro-level, it is possible to extrapolate the problems in the healthcare efficiency. Whether or not these goals are mutually exclusive, it is necessary to find the boundary for their securing.

Hospitals rank among the most important providers of healthcare, making them a significant part of the infrastructure of the economy in developed countries. General tendencies in the development of hospital care have become evident in the past 20–30 years. First, this applies to the optimisation of the number of available beds with respect to the changes in population demography, in terms of the overall structure and efficiency. At the same time, there is a decrease in the average treatment period, caused by the changes in the reimbursement mechanisms in the inpatient care funding, but also by new medical means – equipment as well as new treatment processes. However, reduction in hospital beds in order to increase the system efficiency without maintaining certain contingency capacities may lead to the collapse of the healthcare system at times like the COVID-19 pandemic, when the levels and possibilities of hospital care are both the key and limiting elements to its control. The OECD [5] states in its report that rural areas in developed countries as well as in general usually have half the number of hospital beds per 1000 citizens compared to urban areas. Current experience shows that the lower number of hospital beds as a limiting factor in the management of the pandemic was evident in rural areas where the concentration of persons aged 65+ is usually higher.

From the regional point of view, the structure of the hospital care provider network is generally differentiated. Large cities mostly show higher concentration of hospitals and specialised centres, the care in which is consumed irregularly depending on acute care. On the other hand, free movement of patients without specified rules for the system permeability affects hospitals in large cities. Suitable, optimal ratio of healthcare professionals providing high-quality care to the citizens is essential for the efficient functioning of hospitals. It is necessary to know the development trends in the hospital sector, which are likely to vary significantly in the individual regions. The above is also confirmed by published researches. Vrabková, Vaňková [6] detected technical efficiency of the Czech hospitals, with the associated disparities caused by various factors, including regional factors. Gavurová et al. [7] pointed out regional disparities in the technical equipment of hospital care in Slovakia. Sendek [8] outlined the technical efficiency of selected Slovak hospital in the context of introduction of new technologies.

This article focuses on the modelling of productivity of the capacities and dynamics of hospital care in the Czech Republic and the Slovak Republic. The modelling of technical efficiency is aimed at the selected aggregated input and output parameters for the specific territorial self-governing units, i.e., regions. It is obvious that international comparison entails certain risks and partial inaccuracies. However, the reporting of healthcare statistics is very similar in both countries. Certain modifications were made, so that the reporting of the number of available beds in hospitals respects the terminology of reported indicators in the Czech Republic.

The article aims to identify the trends and disparities in the productivity of the capacities of hospital care on the regional level (NUTS III.) in the Czech Republic and the Slovak Republic in the last decade (2009–2018) before the COVID-19 pandemic using the multi-criteria decision methods.

Three research questions (RQ1–RQ3) were formed to support the objective:

- **RQ1**: Are the differences in the trends of technical efficiency in the capacities of hospital care in the individual regions smaller in the Czech Republic compared to the Slovak Republic?

This research question is based on the assumption that in the reference period, the numbers of beds were significantly reduced in total as well as in certain regions of the Slovak Republic compared to the regions of the Czech Republic, yet the outputs (numbers of inpatients and treatment days) did not drop proportionately, and so it can be assumed that Slovak regions would show better results in terms of the efficiency dynamics.

- **RQ2**: Are the worst results of average technical efficiency and productivity between 2009 and 2018 reported in the regions where the capitals of the selected countries are located and in the regions with multiple large cities?

The second research question is based on the assumption that the differences in the development of technical efficiency in the regions of both countries are negatively influenced by the presence of the largest cities (capitals) and a greater number of large cities as centres of highly specialised care of national importance are located there.

- **RQ3**: How the technological conditions delimited by the production limits influence improvement/deterioration of technical efficiency of the capacities of hospital care on the regional level?

The third research question is based on the assumption that beds, as one of the three selected inputs, significantly influence the evaluation of technical efficiency. The numbers of beds in the regions of both countries are the result of healthcare policies, which attempted in the reference year to increase the utilisation (productivity) of hospital beds. This takes effect in the technological conditions (frontier shift, FS) rather than in the efficiency change (EC).

The fundamental method for the evaluation of technical efficiency of the regions is the DEA model, followed by the window analysis and the Malmquist Index.

The article has five parts. The first part is this introduction. Part two focuses on synthesising the knowledge for the use of the DEA method and window analysis in healthcare. The third part deals with the research methodology including the statistical description and basic dynamics of the individual input and output parameters. The results of analysis are presented in part four. And the last part incudes evaluation of the results, conclusion, and discussion about the problem in relation to the results attained.

### Literature review: using the DEA method and the window analysis in the healthcare system

A number of specialist articles and publications examined the use of the DEA method for the evaluation of technical efficiency of homogeneous production units. The first DEA model was proposed by Charnes, Cooper, Rhodes [9]. Called the CCR model, it uses optimisation calculation to calculate the weights of inputs and outputs, while relying on the assumption of constant returns to scale. By contrast, Banker, Charnes, Cooper [10] proposed modification of this model which uses variable returns to scale. In healthcare, the DEA model and its variants have been favoured for the evaluation of technical efficiency of hospitals, outpatient clinics and other wards, but also regions, representing the evaluated set of DMUs in this research. The article by Kohl et al. [11] is proof that the healthcare sector is one of the main areas of use of the DEA model. The study reviewed 262 articles focusing of the evaluation of efficiency of healthcare units. To evaluate efficiency of production units in time, dynamic models may be used as they allow evaluation of changes in the sectoral production technologies. In terms of efficiency in the public sector, it is possible to extend the concept of technology to the frontier shift factor of production means for the technology and a part that contains the change in the objectives, motivations, and regulations. The vast majority of authors use technical parameters to assess the efficiency, but there are sporadic articles where the authors include qualitative indicators in the output parameters of the process model [12, 13]. Defining healthcare quality as one of the output variables in the DEA model has sparked discussions and interest among the economists in general.

Hospitals and their departments, as DMUs, were evaluated in a number of studies. The micro-level was addressed, for instance, by [11, 14, 15], while the meso-level was evaluated in the studies of Kočišová et al. [16] and Štefko et al. [17], to name a few. Several publications also focused on the macro-level, i.e., the international evaluation of healthcare systems in the individual countries [18–21].

With respect to the orientation of inputs and outputs and the distinctive nature of the returns to scale, the individual DEA models usually focus on the evaluation of one-year periods, not showing the trends and impacts of technological changes, e.g., regulation in the healthcare system. Dlouhý, Jablonský, Zýková [22] add that administrative regulations in healthcare dictate the level of production. Quantitative tools for this evaluation include, for instance, the window analysis and the Malmquist Index. The window analysis is suitable for detecting the trends in efficiency, while the Malmquist Index allows evaluation of multiple inputs and outputs in physical units. As specified by Ozcan [23], “compared to the Malmquist Index, window analysis is a more straightforward approach as the efficiency of DMUs is evaluated only within their respective years, then various periodic averages are calculated to observe the overall trends in performance.” The use of these selected methods is not so frequent, and a number of articles rather focuses on the resulting aspect, i.e., the production unit ranking according to the efficiency attained over time, not reflecting so-called post-optimisation analysis.

Weng, Blackhurst, Mackulak [24] expanded the classic DEA model with the window analysis, taking into account the production unit efficiency over the defined period of time. The evaluation focused on 65 hospitals providing both acute care and aftercare over a 5-year period. The modelling of technical efficiency was made within the two-, three-, and four-year time window. Finally, they used the Malmquist Index to verify the time productivity of the selected hospitals and the efficacy of the proposed approach.

Non-parametric DEA method was used to analyse Greek hospitals over a 5-year period by Flokou, Aletras, Niakas [25]. The efficiency trend was evaluated by the Window-DEA method that allows annual comparison of the results. The authors used a two-year time window and selected the following input parameters: the number of beds, the number of physicians, and the number of other medical staff. There were three output parameters: the number of hospitalisations, the number of surgeries, and the number of outpatient visits. Finally, they used the Malmquist Index to evaluate the productivity of the individual hospitals between the reference periods.

Jia, Yuan [26] used the Window-DEA method to evaluate the changes in the operation of public hospitals using the seven-year data set of indicators. The analysis showed that during the reference period, the operational efficiency of the hospitals tended to the increase in efficiency, followed by a temporary drop in efficiency shortly after the introduction of changes (establishment of hospital branches).

The efficiency of hospitals, using the window analysis in 2011–2016, was also explored by Fuentes et al. [27]. Their study was focused on acute-care public hospitals in Spain. They chose the time window analysis, as this expansion of the Data Envelopment Analysis allows comparison of efficiency of a small number of homogeneous units over a specified period of time, as well as analysis of changes in efficiency in time. The analysis concluded that the average efficiency of hospitals was very good. Nevertheless, they proposed specific measures to increase the performance of these hospitals.

Regional disparities have become an increasingly important constraints to the growth, as described by Štefko et al. [17]. Evaluation of regional efficiency of healthcare facilities during 2008–2015 using the Window-DEA method concluded that there is an indirect relationship between the variables in time and the results of the estimated efficiency in all regions of the Slovak Republic. The authors used a 4-year window for the evaluation.

A new approach to the evaluation of performance of the hospitals was introduced by Ghahremanloo et al. [28]. Their case study evaluated 11 hospitals over a three-year period. The new DEA-EEP model evaluates the efficiency, efficacy, and productivity of the hospitals at once. The results showed that most hospitals tried to improve the quality of their services thanks to systematic changes introduced in the healthcare policy level.

In their article, Miszczynska, Miszczyński [29] focused on the evaluation of efficiency of the Polish healthcare system between 2013 and 2018. Their analysis was based on the output-oriented DEA model and the analysis of time windows with the window width of 2 years. The analysis was completed with a determination of the source of productivity changes and factors that influence efficiency. Through modelling, it was found that the efficiency of the healthcare system is influenced especially by the number of medical staff, accreditation certificate of the healthcare facility, and the wating time for medical services.

The above approaches to the evaluation of efficiency trends using the window analysis and Malmquist Index have also been used in this research, complemented with the statistical and regression analyses of the results in order to determine various dependency rates between the variables and the technical efficiency results.

## Methods

**Fig. 1:**
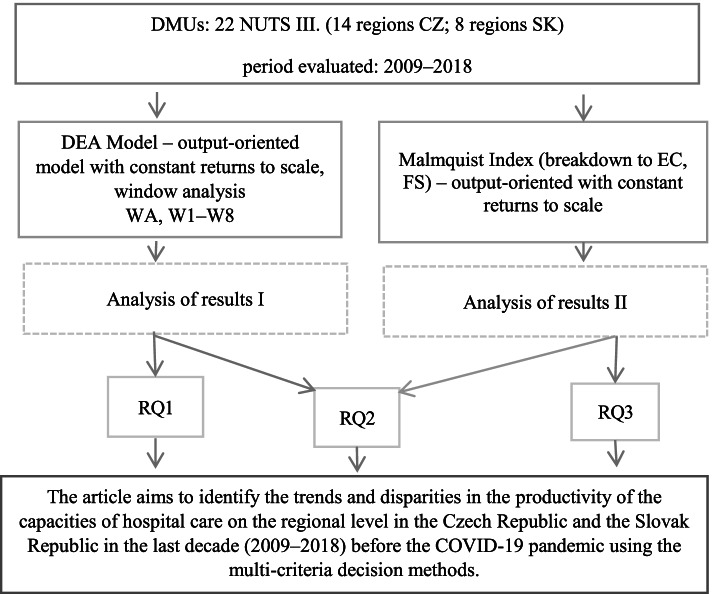
Research methodology

The research methodology is outlined in Fig. 1 and then specified in the subchapters below. The methodology was chosen in accordance with the objective set and the three research questions.

### Regions and the selected technical parameters of hospital care

The provision of inpatient hospital care in the Czech Republic was compared to the Slovak Republic in terms of the aggregated data according to NUTS III (regions). The states were selected for a number of reasons. First, both were part of a single country in the past, a fact that determines certain common systemic elements for this international evaluation. Both countries have the same healthcare system based on the public health insurance, so-called Bismarck healthcare model. Moreover, these neighbouring Central European states use the same administrative division of public administration – state, regions, municipalities. This means, the regions of the Czech Republic and the regions of the Slovak Republic fall within NUTS III category of the European Nomenclature of Territorial Units for Statistics. An indicator which is not the same is the population size – the Czech Republic has roughly twice the population. The selected input and output parameters were therefore converted to 10,000 citizens.

Data for the Czech Republic were primarily taken from the Czech Health Statistics Yearbook for 2009–2018 [30], published by the Institute of Health Information and Statistics of the Czech Republic (IHIS CR), with data for hospital care, i.e., both outpatient and inpatient. Data from the same area for the Slovak Republic come from the statistical documents Health Statistics Yearbook for 2009–2018 [31] and Bed Fund 2009–2018 [32], published by the National Health Information Centre of the Slovak Republic (NHIC SR). To make the data comparable and increase their explanatory power, they were converted using the methodology of inpatient care reporting utilised in the Czech Republic. The reference period was the last decade before the COVID-19 pandemic.

Until 1990, the structure of the healthcare facility network was identical in the Czech Republic and in the Slovak Republic, a result of the previous common existence in a single country [33]. Following the split, both countries implemented fundamental restructuring as well as privatisation of healthcare facilities. The regulated decrease in the number of acute inpatient beds started in 1997 in favour of the aftercare beds, the need of which was growing due to population ageing. The implementation of changes in the area of provision of healthcare, the funding of healthcare (especially the introduction of the DRG classification system), and the availability of healthcare influence the average duration of hospitalisations, the use of hospital beds, as well as the overall equipment of the hospitals.

The total of 32,065 healthcare facilities (including field offices) were registered in the Czech Republic as of the end of 2018. Inpatient care was provided by 314 facilities, thereof 194 hospitals, with the total capacity of 60,633 beds. The number of hospitals did not change significantly compared to 2009 (increase by 3 hospitals), but the role of the hospital promoters and owners of the hospital assets changed, caused by the reform in the public administration as a whole. As of the end of 2018, the total of 12,902 healthcare facilities were registered in the Slovak Republic. Of this number, 180 facilities provide their services in the form of inpatient care. The inpatient care network comprises general hospitals, specialised hospitals, spa centres, treatment facilities, hospices, and nursing homes. The number of hospitals in the Slovak Republic remained almost stable within the reference decade. As of 31 December 2018, there were 114 hospitals with the capacity of 29,863 beds; the number decreased by 4 hospitals against 2009.

The set of units examined are regions as higher territorial self-governing units of the selected countries. The data comprise all hospital care providers regardless of the type or legal form. The modelling of the technical efficiency was performed using the output-oriented model, which is based on the assumption of constant returns to scale. This model should reduce the output parameters in order to attain the target, that is to make the given homogeneous production unit efficient with respect to the defined input parameters. The number of beds, the number of physicians, and the number of general nurses were chosen as input parameters. The number of beds of a healthcare facility specifies the capacity of the inpatient care and is an important regional-level input indicator, as documented by the results of the systematic scoping review [34]. Human resources in healthcare implement the medical care, bringing new and innovative medical treatments that influence the condition and quality of health of the patients, as introduced by Vrabková, Vaňková [35]. Likewise, the articles by Trebble et al. [36], Winkelmann et al. [37] point out these key regional-level parameters and emphasise the need to optimise the staffing resources in healthcare within the region. The number of hospitalised patients and the number of treatment days were chosen as output parameters. The output parameters were chosen in a manner to correspond to the logic of the selected inputs while allowing their monitoring in an aggregated form on the level of the individual regions. The productivity and structure of inpatient units is usually referred to the number of treatment days and the number of hospitalised patients, as also documented by the results of Jia, Yuan, 2017 [26] and Bouckaert et al., 2018 [38]. Financial parameters were deliberately not included in the input and output parameters, as the research focuses on key personnel and technical capacities allocated in the regions in order to ensure hospital care and guaranteed by the respective regional governments. Financial parameters could also distort the results of technical efficiency of the capacities because hospital care in the regions is implemented in varying proportions by both private and public providers and with mixed cash flows. The definition and description of the individual parameters is introduced in Table 1 below.

**Table 1:** Definition of variables

Indicators		Definition
×1	Number of physicians	Professionally competent physicians under professional supervision; professionally competent physicians without professional supervision; physicians with specialised competence.Data as of 31 December, converted number of workloads per 10,000 citizens.
×2	Number of nurses	General nurses; specialist assistants; paediatric nurses. Data as of 31 December, converted number of workloads per 10,000 citizens.
×3	Number of beds	Specified number of beds as of the last day of the reference period, i.e., 31 December, per 10,000 citizens.
y1	Hospitalised patients	Non-additive data. Calculated as the average number of admitted and discharged patients in the reference period. Converted number per 10,000 citizens.
y2	Number of treatment days	Whole day during which the patient is provided with medical services offered by the healthcare facility, i.e., including accommodation and food. The first and the last calendar day spent in the healthcare facility count as full treatment days. Converted number per 10,000 citizens.

The statistical characteristics of the selected parameters (inputs and output) are documented in Table 2, showing minimum, maximum, and mean values. The last two lines express the absolute average increase/decrease and the mean coefficient of increase of the inputs and output per the regions in the given country between 2009 and 2018. From the point of view of mean values, it is evident that the regions of the Czech Republic show a significantly higher number of physicians (× 1) and nurses (× 2) and a slightly higher number of beds compared to the regions of the Slovak Republic. Likewise, the outputs (y1, y2) are higher in the Czech regions. The dynamics values $\left(\overline{d},\overline{k}\right)$ report, in the period of view (2009–2018), an increase in the number of physicians (× 1) and in the number of hospitalised patients (y1) in both countries, more significantly in the Czech regions. The number of nurses (× 2) increased in the Czech regions, while a slight decrease was reported in the Slovak regions. The number of beds (× 3) decreased in both countries during the reference period, but this decrease was much slighter in the Czech regions (mean decrease by 72 beds per region) than in Slovakia (mean decrease by 404 beds per region).

**Table 2:** Statistical characteristics and basic dynamics of inputs and outputs between 2009 and 2018

		×1	× 2	× 3	y1	y2
Min.	CZ	321	1055	1187	51,830	300,071
	SK	344	981	1931	65,452	116,360
Max.	CZ	4650	11,994	10,233	345,827	2,560,682
	SK	1491	3195	5740	170,911	1,356,790
Mean	CZ	1445	4190.5	4256	157,182	1,077,624
	SK	760	2064.6	3870	122,989	920,674
$\overline{d}$	CZ	359.1	498.9	−71.8	6629.2	− 156,199.2
	SK	143.7	−98.3	− 403.9	4150.7	− 106,730.8
$\overline{k}$	CZ	1.02	1.01	1.00	1.00	0.99
	SK	1.02	0.99	0.99	1.00	0.99

Specific results of the above statistical description and basic dynamics for the individual regions are introduced in Additional file 1, Table I.

The individual regions in both countries (CZ: 14 regions; SK: 8 regions) have been differentiated in terms of population, with the contributing factor of settlement structure, i.e., presence of large cities (regional capitals) and capitals of the country. In the Czech Republic, such regions include the Capital City of Prague CZ010, the South Moravian Region CZ064, and the Moravian-Silesian Region CZ080; and in the Slovak Republic, these are the Bratislava Region SK010, the Prešov Region SK041, and the Košice Region SK042.

### Methods: DEA CCR and window analysis

Evaluation of production units was performed in three sequential steps. The output-oriented DEA model with constant returns to scale, so-called CCR model, was chosen for the evaluation of efficiency of inpatient care in the individual regions. The mathematical expression of this model is as follows (1):

1 $$\text{minimise under conditions}\hspace{0.12em}\begin{array}{lc}g=\sum\nolimits_j^mv_jx_{\mathit{jq},}&\\\sum\nolimits_i^ru_iy_{\mathit{ik}}\leq\sum\nolimits_j^mv_jx_{\mathit{jk},}&k=1,2,\dots,n,\\{\textstyle\sum_j^r}u_iy_{iq}=1&\\u_I\geq\varepsilon&I=1,2,\dots,r,\\v_I\geq\varepsilon,&j=1,2,\dots,m\end{array}$$

where: u_i_ is the weight given to output *i*, *y*_*iq*_ is the amount of output *i* produced by DMU *q, v*_*j*_ is the weight given to input *j, x*_*jq*_ is the amount of input *i* produced by DMU *q.*

The optimal value of the purpose function is U_q_ ≥ 1. The degree of technical efficiency is given by the ratio of the weighted sum of inputs to the weighted sum of outputs, but weights are sought such that the value of the efficiency measure is equal to or greater than one. A value of 1 is therefore assigned to effective units, a value greater than 1 to inefficient units.

The subsequent step of the evaluation was calculation of the window analysis (WA) for the reference period (2008–2019). It is fundamental for the purpose of analysis to determine the time window duration [39]. A three-year time window was chosen, as the authors deem it very important to detect the time trends within the structure of healthcare provided and to attain statistical stability of the estimates obtained.

The total number of windows *w* in the solved problem can be expressed by the following relationship:

2 $$w=k-p+1$$

The following applies:

3 $$\mathrm{number}\ \mathrm{of}\ \mathrm{DMUs}\ \mathrm{in}\ \mathrm{each}\ \mathrm{window}:\mathrm{np}/2$$

4 $$\mathrm{number}\ \mathrm{of}\ \mathrm{different}\ \mathrm{DMUs}:\mathrm{npw}$$

where: w = number of windows; n = number of DMUs; k = number of periods; p = duration of window (p ≤ k).

The total of 66 production units were analysed in each of the eight windows for *n* = 22 production units, in T = 10 consecutive periods with the defined window width w = 3. This means 24 efficiency rates were calculated for each of the 22 production units. For the purposes of the final calculation, the arithmetic mean of all values determined was calculated using the formula (5).

The total efficiency rate for the reference period is given by the relationship:

5 $${E}_q=\frac{\sum_{i=1}^z\sum_{t=1}^w{E}_{iq}^t}{z.v},\kern4.5em q=1,2,\dots, n$$

The last step in the evaluation of the production units in time is the Malmquist Index (MI) and its breakdown. When evaluating the changes of efficiency in time (dynamic approach to technical efficiency), the MI allows its breakdown into two components: (i.) changes in the relative efficiency of the units against the set of the remaining units, and (ii.) technology-induced frontier shift of the production possibilities [40, 41].

The construction of the MI is based on the assumption that evaluation focuses on the production units of a certain branch over the period of time *t = 1, 2, ..., T.* For each period, technology *S*^*t*^ is known, through which inputs *x*^*t*^ are transformed into outputs *y*^*t*^. The function *Dq*^*t*^ *(x*^*t*^*, y*^*t*^*)* characterises the technology in time *t* and allocates the efficiency rate to the production unit evaluated *U*_*q*_. Efficient units define the frontier of production possibilities. The function ${D}_q^{t+1}$ (*x*^*t*^*, y*^*t*^) correlates the inputs and outputs from the period *t* with the technology from the period *t + 1*, while the function ${D}_q^{t+1}$ (*x*^*t* + 1^*, y*^*t* + 1^) correlates the inputs and outputs from the period *t + 1* the technology from the period *t*. However, a situation may occur where (*x*^*t* + 1^, *y*^*t* + 1^) does not belong to the technology *S*^*t*^, there can be a case ${D}_q^t$ *(x*^*t*^*, y*^*t*^*)* > *1*, i.e., the unit evaluated attains efficiency which is higher than the frontier of production possibilities in the previous period. Also, opposite situation may arise where ${D}_q^t$ (*x*^*t*^*, y*^*t*^) < *1* if the course of production possibilities decreases compared to the previous period [42].

The mathematical expression of the Malmquist Index is as follows (6):

6 $${M}^Q\left({x}^t+1,{y}^t+,{x}^t,{y}^t\right)$$

where *E*_*q*_ is the change of the unit’s relative efficiency *q* relative to other units between the periods *t* and *t + 1*, *P*_*q*_ describes the change of the frontier of production possibilities due to the technology development between the periods *t* and *t + 1*. Mathematical representation of the components *E*_*q*_ and *P*_*q*_ is (7) and (8):

7 $${E}_q=\frac{D_q^{t+1}\left({x}^{t+1},{y}^{t+1}\right)}{D_q^t\left({x}^t,{y}^t\right)}$$

8 $${P}_q=\left[\frac{D_q^t\left({x}^{t+1},{y}^{t+1}\right){D}_q^t\left({x}^t,\kern0.5em {y}^t\right)}{D_q^{t+1}\left({x}^{t+1},{y}^{t+1}\right){D}_q^{t+1}\left({x}^t,{y}^t\right)}\right]\frac{1}{2}$$

For the purposes of the MI, where there are tasks with multiple inputs and outputs, it is necessary to use a certain DEA model, for instance, the CCR model specified above, which envisages constant returns to scale. The breakdown of the MI allows expressing its two components (*efficiency change and frontier shift*), where *MI = efficiency change (E*_*q*_*) x frontier shift (P*_*q*_*).*

In case of the output-oriented MI, the results are interpreted as:

- MI_(output)_ > 1 (improves);
- MI_(output)_ = 1 (remains unchanged);
- MI_(output)_ < 1 (decreases) [6, 23, 40, 43].

## Results

### Results: windows analysis

The window analysis is based in the use of moving averages. One of the important elements is so-called time window (W1–W8) because several individual calculations of efficiency rate are made within the WA. The number of calculations corresponds to the number of windows allocated to the initial period (2009–2018), i.e., eight windows. The windows overlap and have the same width of three years: W1 2009–2011, W2 2010–2012, W3 2011–2013, W4 2012–2014, W5 2013–2015, W6 2014–2016, W7 2015–2017, W8 2016–2018.

The modelling of technical efficiency was made using the output-oriented model that compares the regions in terms of the necessary increase of the outputs to attain efficiency. In output-oriented models, an efficient unit = 1, while an inefficient unit > 1. An improvement within the output-oriented model of production units is a process that leads to the increase of certain or all output values (y1 and y2). The output-oriented model with constant returns to scale (OO_CRS) was also chosen for the evaluation. A classic economic concept, the returns to scale describe the change in the output after a proportional change in the inputs. Constant returns to scale can be interpreted as a directly proportional change in the number of outputs based on the change in the number of inputs [23, 43].

The aggregated results for the 22 regions (14 R_CZ and 8 R_SK) are shown in Table 3, and broken down to the regions in Additional file 1, Table II. Table 3 contains the total results (Total) and the results for the individual countries (R_CZ and R_SK). In the Czech regions, the overall best average result was attained within W5, while in the Slovak regions, the overall best average result was attained within W7. It is also evident that on average, the Slovak regions attained better efficiency results than the overall average.

**Table 3:** Aggregate results of the windows analysis

		W1	W2	W3	W4	W5	W6	W7	W8	WA
R_CZ	Mean	1.049	1.054	1.073	1.050	**1.045**	1.049	1.071	1.080	1.059
	Median	1.050	1.055	1.074	1.045	1.049	1.040	1.065	1.063	1.051
	SD	0.033	0.030	0.026	0.024	0.026	0.031	0.045	0.059	0.027
	VR	0.106	0.106	0.095	0.080	0.093	0.130	0.166	0.227	0.095
R_SK	Mean	1.064	1.060	1.052	1.056	1.048	1.043	**1.040**	1.047	1.052
	Median	1.060	1.064	1.047	1.054	1.043	1.044	1.028	1.040	1.052
	SD	0.044	0.040	0.026	0.021	0.038	0.033	0.032	0.032	0.023
	VR	0.133	0.111	0.084	0.082	0.131	0.111	0.100	0.096	0.077
Total	Mean	1.055	1.056	1.065	1.052	**1.046**	1.047	1.059	1.068	1.057
	Median	1.050	1.055	1.068	1.053	1.047	1.043	1.045	1.055	1.051
	SD	0.038	0.034	0.028	0.023	0.031	0.032	0.044	0.053	0.026
	VR	0.135	0.111	0.107	0.082	0.132	0.130	0.166	0.227	0.101

Note: VA – Range

**Fig. 2:**
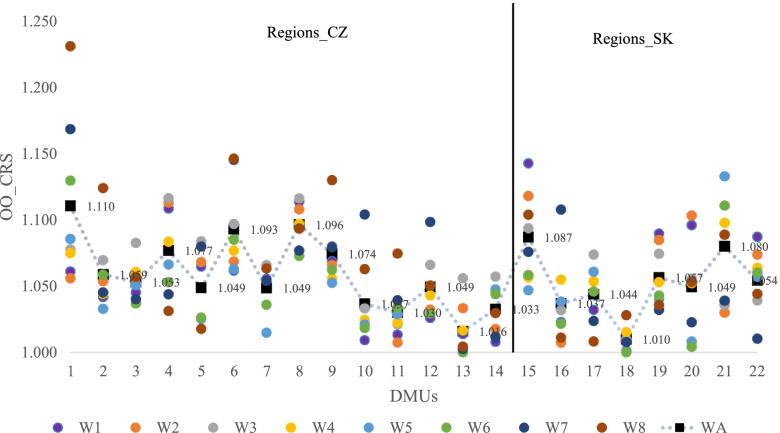
Results of the windows analysis for the individual regions

Figure 2 shows the results for the individual windows (W1–W8) and the results of the windows analysis. These detailed results confirm the aggregate results, i.e., the Slovak regions are slightly better in the individual windows as well as in the overall analysis (WA). However, the differences between the Czech regions and the Slovak regions are not significant. The best region is R18_SK023 (e = 1.010) and the second-best is R13_CZ072 (e = 1.016); both are smaller, rural-type regions in terms of population.

Results of the Czech regions were negatively affected by: the high variance of the results between W1–W8 in R1_CZ 010, the results of regions for W8 2016–2018. In case of the Slovak regions, the results were negatively affected by the results of R15_SK010, the results of regions for W1 2009–2011. In both cases, these are regions with the country capitals, i.e., the largest cities in terms of population.

### Results: Malmquist index

The Malmquist Index (MI) indicates three statuses – improvement, deterioration, and no change in the relative efficiency of a region relative to other regions as of 2018 compared to 2009. The MI consists of two components: the efficiency change (EC) and the frontier shift (FS). The EC expresses the change in the (inner) relative efficiency of a production unit relative to the set of the remaining units; the FS expresses the technology-induced frontier shift of the production possibilities (in case of the healthcare system, these are most likely the changes triggered by public policies and government interventions).

**Fig. 3:**
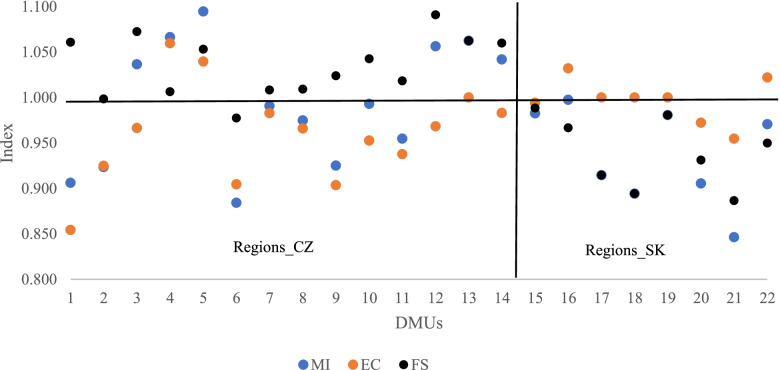
Results of the MI and its breakdown into the EC and the FS

The results attained are shown in Table 4 and Fig. 3. In summary, 73% of regions from the reference set attained an improvement of the relative efficiency in 2018 compared to 2009. From the point of view of the individual countries, improvement was attained in 57% of the Czech regions and in 100% of the Slovak regions. Six regions of the CR deteriorated in 2018 compared to 2009, while the deterioration was influenced by the FS element, i.e., technological conditions.

**Table 4:** Aggregate results for the regions – Malmquist Index (2009, 2018)

*N* = 22 (14, 8)	> 1 (CZ/SK)	= 1 (CZ/SK)	< 1 (CZ/SK)	Mean (CZ/SK)	Median (CZ/SK)	SD (CZ/SK)
MI	6 (6/0)	0 (0/0)	16 (8/8)	0.973 (0.994/0.936)	0.978 (0.992/0.943)	0.066 (0.065/0.050)
EC	4 (2/2)	4 (1/3)	14 (11/3)	0.974 (0.960/0.997)	0.978 (0.966/1.000)	0.047 (0.052/0.023)
FS	12 (12/0)	0 (0/0)	10 (2/8)	1.000 (1.035/0.932)	1.007 (1.033/0.940)	0.057 (0.032/0.036)

Figure 3 shows the results of MI and its two components, EC and FS. DMUs 1–14 are the Czech regions; DMUs 15–22 are the Slovak regions. The diagram makes it clear that the increase in the productivity of regions in 2018 compared to 2009 was influenced in the Slovak regions by the FS component. In contrast, improvement of efficiency EC positively influences the productivity in the Czech regions.

Figure 3 makes it clear that improvement of the MI in the Czech Republic is positively influenced by the EC component. The resulting values of MI productivity was therefore influenced by other factors in both countries.

The correlation analysis and the multiple regression analysis of the individual components of the EC and the FS make it possible to express how the resulting MI value is influenced by these components. The Pearson correlation coefficient *r* proves the positive correlation between: MI and EC = 0.565 (α = 0.005); MI and FS = 0.699 (α = 0.001). It is clear from the above that the increase of the FS value causes the MI value to increase as well, especially in the Czech Republic, where it deteriorates the MI.

Multiple regression according to the enter model, where the FS and the EC are the predictors, and the MI is the dependent variable. The R Square value = 0.999 and the Adjusted R Square value = 0.999 (F = 11,204.405, Sig. F Change = 0.000) specify the extent of variance of the dependent variable (MI) expressed by the se of independent variable (FS, EC). The results show that 99% of variance of the dependent variable is explained by independent variables, which is an excellent result.

The ANOVA was used to verify the null hypothesis, coefficient R^2^ = 0, which was rejected, see Table 5.

**Table 5:** ANOVA test

	Sum of Squares	df	Mean Square	F	Sig.
Regression	0.096	2	0.048	11,204,405	0.000
Residual	0.000	19	0.000		
Total	0.096	21			

## Conclusion and discussion

The research was focused on the technical efficiency trends of the capacities of hospital care on the regional level in the Czech Republic and in the Slovak Republic, where 22 regions were investigated in this regard – 14 Czech regions and 8 Slovak regions – over the period from 2009 to 2018. The research subjects comprised the aggregated inputs and outputs from the individual regions.

The results of the mean technical efficiency in the output-oriented model with constant returns to scale, estimated according to the DEA model and then using the window analysis, did not unequivocally confirm the research questions RQ1 and RQ2. The following was found:

- Regional differences in the productivity in hospital care are slightly greater in the Czech Republic compared to the Slovak Republic;
- On average, the Czech regions are less efficient compared to the Slovak regions; the least efficient is R1_CZ010 (the Capital City of Prague), followed by two medium-sized Czech regions R8_CZ052 (the Hradec Králové Region) and R6_042 (the Ústí nad Labem Region);
- The least efficient Slovak region is R15_SK010 (the Bratislava Region), followed by R21_SK041 (the Prešov Region) and R22_SK042 (the Košice Region).

The research results support the findings of Winkelmann et al. [37] who followed regional distribution of medical staff in Europe on NUTS II level, finding that physicians and general nurses tend to concentrate namely in urban areas to the detriment of rural areas. This concentration of health workforce in large cities, which offer modern living standards including education, is natural and normal, but it affects the quality of care and efficiency of health services. The worst results of efficiency in the set and time in view were indicated in regions with the largest cities in both republics, but on the other hand, these regions provide high-quality, specialised healthcare.

The basic statistical and dynamic analysis and the technical efficiency results including the WA confirm the assumption that better technical efficiency results of the Slovak regions were influenced by the lower number of beds and nurses and by the less pronounced drop in the treatment days compared to the Czech regions.

The evaluation of the mean efficiency and productivity showed that the worst results of the technical efficiency and productivity (deterioration) were reported in regions where the capitals of both countries are located. At the same time, the assumption that regions with the largest cities are less efficient compared to other regions was confirmed in the Slovak regions. This assumption did not materialise in the Czech regions.

Research question RQ3 addressed the issue of technological conditions influencing the provision of hospital care in the regions. The analysis confirmed the following:

- All the Slovak regions improved their productivity (MI) in 2018 compared to 2009, while technological conditions (FS) significantly affected the improvement;
- The Czech regions reported improvement of the productivity in 57% of the regions (8), influenced primarily by the improvement of technical efficiency (EC). Deterioration of the productivity in the remaining 43% of the Czech regions was influenced by the technological conditions (FC).

Results of the Malmquist Index and its breakdown confirm the assumption that the technical efficiency in the provision of hospital care and its improvement in time is primarily influenced by the number of beds and by the number of medical staff. The technological conditions in the regions of both countries are the result of healthcare policies, and the efforts towards the increase of utilisation (productivity) of hospital beds took effect in the reference period, especially in Slovakia.

However, it must be stressed that technical efficiency of the provision of hospital care in the regions is determined by the number of beds, but also by the number of medical staff. The technical efficiency of the provision of hospital care is therefore in conflict with the healthcare quality, a fact that negatively impacts the availability of hospital care, especially in smaller and rural regions, as also noted by Štefko et al. [17]. In this regard, the results are consistent with the conclusions of the OECD report [5] which points out the shortfall in the beds in rural regions, representing a risk factor in the critical, pandemic (COVID-19) periods. They are also in agreement with the new strategy of the Czech Republic, Health 2030 [2], which emphasises the need to ensure stable staffing capacities in healthcare across the regions.

It should be recommended to the state- and regional-level governments to refrain from unilaterally preferring the orientation of public policies on the efficiency of the provision of hospital care, and rather focus on increasing the quality and availability of hospital care, especially in smaller, rural, and border regions, in the interest of population safety during pandemics and other emergencies. The above is accentuated by the fact that the attained results of the evaluation of technical efficiency during 2009–2018 detected technically efficient regions where insufficient capacities (both beds and staff) were reported in 2020/2021 during the COVID-19 pandemic [44, 45].

As part of personnel issues in hospital care, the governments should focus on sustainable capacities of key medical professions, especially primary care nurses and physicians in all regions, primarily those without capitals or large cities with university hospitals because those cities have a much more diversified structures and capacities of hospital care. By contrast, hospital bed capacities must be utilised more efficiently, and it appears appropriate to take advantage of the Slovak practice gained within the Value for Money project.

It must be stated in this respect that planning the optimum number of beds and medical staff is very complicated [34], because it is always necessary to maintain the interrelationship of all forms and kinds of care (e.g., acute intensive inpatient care → inpatient aftercare). In both countries in view, the hospital network and its capacities are ensured and guaranteed by regional governments, and based on the results stated herein, it should be recommended to these governments to cooperate with each other more efficiently in developing these networks, thus ensuring continuity and acute need for healthcare for their citizens, especially during emergency situations or for highly specialised care. The ministries of health as well as the medical expense payers put consistent pressure on improving the efficiency of the healthcare structures provided throughout the territory of these countries, and therefore the regional governments should meet this trend halfway through their proactive approach.

It is also debatable how clients of medical services (patients) will react if they do not find adequate healthcare available in their region. Mafrolla and D’Amico [46] point out that due to insufficient capacities and offer of healthcare, citizens are likely to migrate in order to find more accessible care of higher quality, i.e., “vote by feet”. Regional disparities will deepen, a fact that is not in the interest of any economically developed and democratic country. The finding stated above is supported by the results of a research conducted by Gutiérrez-Hernández and Abásolo-Alessón [47], in which the authors argue that the healthcare sector is a relatively independent and significant regional production sector with a strong potential to create added value and employment, while various levels of productivity of personnel input in healthcare can be observed in EU countries.

Open to debate is how to construe the results of the technical efficiency calculations according to the DEA model, the window analysis, and the Malmquist Index in practice and, above all, in the context of the healthcare quality, which improves with the increase of the medical staff, in contrast to the technical efficiency. One possibility would be expanding the DEA models with quality parameters to the output side, which may bring ambiguous results [45]. Debatable, with respect to the validity of the results of the technical efficiency calculations, is the qualitative nature of the outputs in the form of primary data (results of the patients’ satisfaction surveys), as well as the macroeconomic nature of the outputs in the form of indexes or share indicators (percentage of healthcare revenues per GDP) [20]. Another possibility is the opposite interpretation of the DEA model results, but considering the selected inputs and outputs, i.e., inefficient production units and quality production units. It may be deduced in this respect that higher quality of hospital care in the Czech Republic and the Slovak Republic alike is ensured by regions where the country capitals are located, including the fact that state-of-the-art healthcare centres, university hospitals and other pivotal healthcare facilities are concentrated there.

## Supplementary Information

**Additional file 1.**

## Data Availability

The datasets generated and/or analysed during the current study are available on the website of the Institute of Health Information and Statistics of the Czech Republic (https://www.uzis.cz/index.php?pg=vystupy%2D%2Dknihovna&id=275) and the website of the National Health Information Centre (https://www.nczisk.sk/Statisticke_vystupy/Tematicke_statisticke_vystupy/Postelovy_fond/Pages/default.aspx) and (https://www.nczisk.sk/Statisticke_vystupy/Zdravotnicka_rocenka/Pages/default.aspx).
