# Supplementary material for: Productivity analysis of regional-level hospital care in the Czech republic and Slovak Republic
Source: BMC Health Serv Res. 2022 Feb 11;22:180. doi: 10.1186/s12913-022-07471-y (PMC8840586; doi:10.1186/s12913-022-07471-y)
Supplement: Supplementary file 1 — Additional file 1. [file 12913_2022_7471_MOESM1_ESM.docx]

**Additional file 1**

**Table I.** Absolute increases/decreases in the inputs and outputs during 2009–2018

| NUTS III. | Inputs | | | Outputs | |
| --- | --- | --- | --- | --- | --- |
|  | x1 | x2 | x3 | y1 | y2 |
| **CZ010** | 48.1 | 42.2 | -57.8 | -1 665.0 | -49 011.0 |
| CZ020 | 40.4 | 67.0 | 49.2 | 1 354.6 | 299.8 |
| CZ031 | 15.2 | 37.8 | -18.0 | 1 221.8 | -5 387.7 |
| CZ032 | 12.1 | 16.7 | -22.1 | 916.3 | 1 305.8 |
| CZ041 | 10.1 | -3.1 | -52.1 | -350.9 | -9 759.0 |
| CZ042 | 16.0 | 11.0 | -3.2 | -290.9 | -21 434.1 |
| CZ051 | 10.9 | 12.5 | -29.1 | 255.3 | -7 184.8 |
| CZ052 | 7.1 | 24.1 | -22.8 | - 85.4 | -7 902.7 |
| CZ053 | 23.4 | 16.3 | -17.4 | -91.2 | -10 186.0 |
| CZ063 | 24.5 | 41.5 | -12.6 | 612.9 | -7 897.9 |
| **CZ064** | 56.7 | 75.3 | -50.2 | 702.3 | -26 379.9 |
| CZ071 | 34.0 | 57.4 | -2.4 | 1 578.2 | -5 006.7 |
| CZ072 | 15.1 | 33.2 | -27.4 | 612.3 | -3 145.4 |
| **CZ080** | 45.5 | 66.8 | -30.0 | 2 058.9 | -4 509.7 |
| **SK010** | 26.3 | 6.2 | -56.6 | -777.1 | -14 611.9 |
| SK021 | 3.6 | -32.3 | -63.3 | -1 088.6 | -12 707.6 |
| SK022 | 5.4 | -13.7 | -27.2 | - 466.1 | -5 655.0 |
| SK023 | 18.4 | -7.3 | -59.9 | - 886.8 | -16 709.4 |
| SK031 | 15.3 | -5.8 | -27.8 | -27.3 | -8 988.0 |
| SK032 | 28.4 | 6.8 | -57.0 | -411.0 | -14 748.4 |
| **SK041** | 26.5 | -18.3 | -84.4 | -318.9 | -24 814.8 |
| **SK042** | 19.9 | -34.0 | -27.7 | -174.9 | -8 495.7 |

**Table II.** Mean efficiency of the Czech and Slovak regions during 2009–2018

| DMUs | | Average efficiency rate | Order | Stability | |
| --- | --- | --- | --- | --- | --- |
|  |  |  |  | $S_{n}^{2}$ | *R* |
| **R1_CZ010** | **Capital City of Prague** | **1.1104727097** | **22.** | **0.004825571** | **0.21067** |
| R2_CZ020 | Central Bohemian Region | 1.0587124447 | 15. | 0.001299352 | 0.12246 |
| R3_CZ031 | South Bohemian Region | 1.0531118417 | 12. | 0.000417720 | 0.07639 |
| R4_CZ032 | Plzeň Region | 1.0769675918 | 17. | 0.001328993 | 0.13090 |
| R5_CZ041 | Karlovy Vary Region | 1.0488685412 | 8. | 0.001216432 | 0.12105 |
| R6_CZ042 | Ústí Region | 1.0929065021 | 20. | 0.001709517 | 0.13677 |
| R7_CZ051 | Liberec Region | 1.0485647306 | 7. | 0.000965087 | 0.09505 |
| R8_CZ052 | Hradec Králové Region | 1.0963291024 | 21. | 0.000476307 | 0.08097 |
| R9_CZ053 | Pardubice Region | 1.0743420642 | 16. | 0.001024968 | 0.14764 |
| R10_CZ063 | Vysočina Region | 1.0366956721 | 5. | 0.001759377 | 0.18667 |
| **R11_CZ064** | **South Moravian Region** | **1.0300950691** | **3.** | **0.000785621** | **0.09995** |
| R12_CZ071 | Olomouc Region | 1.0494482988 | 11. | 0.001147813 | 0.12882 |
| R13_CZ072 | Zlín Region | 1.0158671894 | 2. | 0.000857396 | 0.11752 |
| **R14_CZ080** | **Moravian-Silesian Region** | **1.0326865248** | **4.** | **0.000487331** | **0.07287** |
| **R15_SK010** | **Bratislava Region** | **1.0869277899** | **19.** | **0.001446458** | **0.12417** |
| R16_SK021 | Trnava Region | 1.0369034107 | 6. | 0.001520395 | 0.12816 |
| R17_SK022 | Trenčín region | 1.0369034107 | 7. | 0.001520395 | 0.12816 |
| R18_SK023 | Nitra Region | 1.0439300000 | 1. | 0.001123548 | 0.09793 |
| R19_SK031 | Žilina Region | 1.0098900000 | 14. | 0.000215813 | 0.04390 |
| R20_SK032 | Banská Bystrica Region | 1.0565600000 | 10. | 0.000654264 | 0.07977 |
| **R21_SK041** | **Prešov Region** | **1.0493600000** | **18.** | **0.002516568** | **0.18125** |
| **R22_SK042** | **Košice Region** | **1.0800300000** | **13.** | **0.003465853** | **0.19551** |

*Key:* $S_{n}^{2}$ *– variance; R – range of variation*
